# Consent, ethics and genetic biobanks: the case of the Athlome project

**DOI:** 10.1186/s12864-017-4189-1

**Published:** 2017-11-14

**Authors:** Rachel Thompson, Michael J. McNamee

**Affiliations:** A-STEM, College of Engineering, Swansea University, Crymlyn Burrows, Fabian Way, Swansea, SA1 8EN UK

**Keywords:** Biobank, Sports genomics, Research ethics, Consent, International governance, Athlome Project Consortium, Data sharing, Regulation

## Abstract

This article provides a critical overview of the ethics and governance of genetic biobank research, using the Athlome Consortium as a large scale instance of collaborative sports genetic biobanking. We present a traditional model of written informed consent for the acquisition, storage, sharing and analysis of genetic data and articulate the challenges to it from new research practices such as genetic biobanking. We then articulate six possible alternative consent models: verbal consent, blanket consent, broad consent, meta consent, dynamic consent and waived consent. We argue that these models or conceptions of consent must be articulated in the context of the complexities of international legislation and non legislative national and international biobank governance frameworks and policies, those which govern research in the field of sports genetics. We discuss the tensions between individual rights and public benefits of genomic research as a critical ethical issue, particularly where benefits are less obvious, as in sports genomics. The inherent complexities of international regulation and biobanking governance are challenging in a relatively young field. We argue that there is much nuanced ethical work still to be done with regard to governance of sports genetic biobanking and the issues contained therein.

## Background

Biobanking sits at the intersection of many research disciplines and involves heterogeneous types of actors including legislators, policy makers, research participants, researchers and funders. It raises issues of private and public interests, protection of individuals and the development of research that may have population wide benefits [1]. This complexity means that nuanced ethical and regulatory work is needed to provide quality governance and an ethically responsible environment for the conduct of sports genetic biobanking activities. We present here an overview of the ethics and governance of biobank research with reference to the Athlome Consortium [2] as an example of large scale internationally collaborative sports genomic biobank research.

First, we give an overview of biobanks. Secondly, there is a discussion of the traditional account of informed consent and possible alternative consent models applicable to biobank research, including verbal consent. Thirdly, there is brief consideration of legislation and governance frameworks applied to biobanking, with particular attention to the challenges of international collaboration. There are many ethical issues arising from the use of biobanking [3]. These include privacy, confidentiality, security and respect for autonomy. The importance of ethical concerns with regard to biobanking and genomic research is underlined by the UNESCO International Bioethics Committee (IBC): ‘…the human genome is one of the premises of freedom itself and not simply raw material to manipulate at leisure’ [4].

Of the several ethical issues, only consent is addressed here in detail. We do not make a detailed exploration of the cognate functions of consent such as protecting anonymity and confidentiality as these are beyond the scope of the present investigation. Nevertheless, consent is widely considered as the most broad ranging and significant overarching concept that ties together many ethical concerns and is central to consideration of the ethics of biobank research practices. Moreover, researchers and clinicians often fail to understand the different models of consent and their ethical nuances. In order that subjects, participants and patients offer their valid consent, researchers must consider which model of consent they will operate with and have a clear justification for it. To this end we articulate six different models of consent, and one competing model referred to as “waived consent” in the context of genomic biobanking.

Biobank research has rapidly grown in both scale and areas of application. Carefully governed it offers potential benefits both predicted e.g. drug targeting, personalised treatment, disease risk prediction, and those we cannot yet envisage. Ensuring the integrity of such research whilst protecting participant interests is fundamental if researchers are to fully explore all the possibilities of biobanking and genomics. The complexity and ‘unknown’ elements of these approaches require deep consideration of underlying norms and the development of governance that reflects the issues raised.

## Biobanks

There is no universally agreed definition of a biobank [5]. It is, however, widely understood to mean a collection of biological samples that have been taken for the purpose of research. Biobanks are large scale resources that link biological with health or other biographical data. They are often set up for prospective studies, but can also use existing sample collections and are sometimes mixed [5]. Biobanks differ from *biorepositories*, since the latter serve merely as a store for biological samples taken in the run of clinical investigations. Biorepositories store, for example, excess biopsy tissues and blood from testing. Such resources may later be used for research, but they are not usually collected solely for that purpose. Biobank research projects range from disease-specific, for example, the Breast Cancer Campaign Tissue Bank, to population scale, for example, UKBiobank. This heterogeneity raises many challenges for ethics and governance since a one size fits all approach to biobanks may be insensitive to the specific needs of particular biobanks and their participants.

It is a feature of biobanks - being large scale operations -that the sources of the data stored within them will arise from research projects or clinical investigations that themselves have been produced under differing conditions. A significant advantage of the modern biobank over more traditional smaller scale research is the combining of collections to form huge data sets that can be linked with other types of data to investigate a wide range of questions. There is a wide range of potential scientific and social benefits from such research, from how our genetic makeup interacts with environment to drug targeting to sports performance. The use of biobanks to store tissue or genetic material is not new, but the international scale and combination of genotypic with phenotypic data is a recent development. It is this aspect that has generated new normative challenges to researchers and clinicians.

Finally, we attempt to apply these insights to a new biobanking consortium: the Athlome consortium. The Athlome consortium is comprised of pre-existing Athlome projects that each act as a bio guardian to protect the interests of participants in their specific project. It includes collaborative projects with research partners from countries in all developed regions of the world. Ethical responsibility is distributed across the group. Each contributing element designates a researcher/clinician who is understood as a “bioguardian” [2].

Biobank research requires highly complex technological methods for the combining, analysis, sharing and securing of data. There is often uncertainty about the uses of data stored therein. Research questions are generated from analysis of a range of concerns: for example, combining health or performance-related data with genomic data to discover a variant linked to a disease or condition, or patterns of association of particular variants with, for example, susceptibility to injury. The openness and scale of collaborative biobanking sets it apart from traditional exploration of explicit hypotheses in a focused study. This highly technologised and open-ended nature generates ethical ambiguities. These include potential harm from malicious or unintended re-identification, uncertainty of research purpose with implications for consent; and blurring of lines between tissue, data and information, with implications for ownership and access. The complexity of the technical processes involved is a challenge for informed consent.

## Consent

Our current understanding of consent is widely acknowledged to have arisen from critical attention to the governance of clinical research, originating from the Nuremberg Trial principles (Nuremberg Code) [6], and modified by the various declarations of the World Medical Association [7] with the aim of protecting participants in single research projects in a single place. Two fundamental conceptual aspects of the traditional model of consent are voluntariness and informedness [8]. Voluntariness refers to decisions made by potential participants free from coercion or undue influence. Informedness requires the decision maker to have reasonably sufficient information in order to consent or refuse based on that information. Despite the widespread agreement about the general dimensions of the consent (absence of coercion or undue influence; expected benefits, expected risks and safeguards; maintenance of privacy, anonymity and confidentiality; provision of verbal or written information regarding the project aims; right to withdraw without prejudice; and so on) it is nevertheless often a matter of interpretation as to what constitutes a reasonable level of “informedness” [9], or when secondary or tertiary requests to participate are considered robust or coercive. Researchers are required to carefully think through the inherent complexities of the research and the specific competence of participants to understand a particular project or intervention. The older and popular phrase “full and free informed consent” has been exposed as being impossible to fulfill. In principle no-one can know *all* of the possible consequences of any particular research project [10]. What counts as reasonably sufficient and appropriate information is often a matter of interpretation, modulating between the inherent complexities of the research or clinical intervention, and the competence of the subject, participant, or patient (hereforwards “participant”).

For biobank ethics, informedness is a particular concern due to the highly complex nature of the research and the innovative methods involved. Many research projects are constituted by highly specialized teams, the members of which are themselves not expert in all aspects of the technology comprising the research, so it is difficult to see how the public can fully grasp such information to a level required by informed consent. Currently there is no consensus on how much and what type of information is needed for consent to be informed [11]. There is an identified ‘information gap’ between data curators and technicians, researchers and participants [12]. There may be additional cause for concern if only a small number of people understand the systems and risks, and they are not the people explaining participation to donors. Fast moving, highly complex, technology adds to the complexity of the ethical aspects of consent and governance more broadly. This can lead to reactive legislation, or defaulting to the most proscriptive applicable regulation where multiple jurisdictions apply [13]. Under these conditions – a version of the precautionary principle may be applied that is disproportionate – ie more prohibitive than is strictly necessary for the purposes of protecting participant interest. This has been referred to as the “whiplash effect” [3].

More recently there has been a highlighting of consent to a model predicated more on respect for autonomy [10], understood as self governance. It is often transposed under the legal term of “competence” but refers essentially to the capacity of acting in an informed and self-directed manner consistent with one’s goals and aims [8]. The dominant norm for consent is written consent, where suitable surrogates are permissible for those without competence [10].Given that the first wave of bioethical research took the individual patient-clinician relationship as its paradigm, it is worth considering – in the light of biobank development - the extent to which this model is still fit for purpose. It is clear that there are some salient ethical differences between the traditional model and the requirements of research processes that are more epidemiologically focused and lie broadly within a public health oriented model [14, 15].

## Verbal consent

Notwithstanding the norm for first person written consent, in a primarily oral culture, or where participants do not write for some other reason, this may be problematic inter alia for reasons of trust. A global collaborative group such as Athlome, which conducts its research in a variety of contexts not tied to medical institutions like hospitals, will operate across a range of cultural norms. Therefore, it must include adaptive strategies for consent processes in order to reflect and respect differences plus adhere to agreed consortium-wide standards.

Modern audio-video (AV) technology, being mobile and relatively cheap, has the potential to facilitate verbal consent in ways that do not undermine respect for persons within these cultures. Supporting information and updates can be sent to mobile technologies such as smart phones. Alternative means to facilitate verbal consent might be the use of voice over internet protocols and other audio visual technologies (such as Skype, Viber, etc.). It may be possible to video or audio record consent conversations, though this raises the issue of the (somewhat ironic) procedure of the participants’ needing to have consented to being recorded, before being recorded giving their consent. Guidance should be sought from the community, using local research ethics codes and procedures where they exist. This is particularly necessary with indigenous and other potentially vulnerable groups in light of previous unethical conduct by researchers [16, 17]. For example, the San peoples of sub-Saharan Africa have recently produced a research guide which advises on consent and other related matters [18]. There is, however, no need for this to result in a vicious infinite regress.

Consent should be understood as a process rather than a single event (i.e. recording signature and date), a concept widely accepted in longitudinal research [19, 20]. That consent should be understood processally is a notion with serious implications for biobank research given the promise of long term storage and of potential new uses, questions and applications. The traditional paper based consent model is static, implying a ‘one time ask and answer’ that is then filed and (too often) forgotten [20]. Using AV may help to improve ethical consenting of populations that do not primarily communicate in writing, but may also be a method to explore for all populations as it can be inexpensive, simple to use and to store, and can readily be added to over time.

AV may, however, be culturally inappropriate, or no more acceptable than paper-based written consent, for groups where there is a perceived risk to consent being documented. In a culture based on oral communication, there is also a potential trust issue if the researcher requires written or audio recorded consent. That is, the spoken word or verbal assurance may have more power in some orally based cultures than in the West; and therefore it can be perceived that a person’s word is not sufficiently trustworthy if asked a request is made by a researcher to record it. The act of asking can be offensive in and of itself. Therefore, local guidance must be sought prior to beginning consent processes. [16] For non-writing persons in a writing culture, use of mobile technologies has the potential to improve consent quality and to widen access to research to those who may previously not have felt able to participate.

Lack of population diversity is a problem in genetic biobank research [21] and the facilitation of appropriate non-written consent processes may be one way to increase inclusivity, both of a variety of ethnic groups and socially disadvantaged groups within ethnicities. What constitutes diversity itself is contentious since it is not clear that how constructions of race or ethnicity answer the problems. The value of data derived from specific groups’ genomes is such that in the past unethical research practices have taken place, leading to the need for more stringent protection of indigenous and other groups’ rights [17, 18]. Whilst improvement in practices is ethically better for both participants and for continuation of research, we must be alert to ‘ethical colonialism’ and the uncritical imposition of norms of the more economically and technologically developed world on to less powerful communities. There is often high genetic and cultural diversity within a group designated as a particular ethnicity, requiring sensitive and informed approaches to seeking consent and to collection of samples and data. A potential social benefit of genomic research is a better understanding of genetic diversity and how it relates to constructions of ethnicity, but this is beyond the scope of our paper [22].

## Challenges to informed consent

First person, written, informed consent has been seen as the ‘gold standard’ and has been a cornerstone of all recent clinical research [8]. This model is widely perceived to offer the best protection for autonomy and has been transferred from clinical to data based research. For research biobanks, however, which bring together existing data repositories, it is difficult or impossible to gain specific consent, as intended uses of the data are unknown at the time of joining. Not only might uses be unknown, but they may be in principle unknowable at any particular time in the ongoing research processes. A further problem arises in the contexts of retrospective or secondary use consent. Many biobank collections contain samples taken with consent for a particular research use and stored accordingly following that use. There is no universal approach to consent for second uses, other than it should be broadly within original parameters and with research ethics committee oversight. The Public Population Project in Genomics and Society (P^3^G) [23] and Global Alliance for Genomics and Health (GA4GH) [24] have developed guidance on assessing ‘legacy collections’ and consent which may assist Athlome in deciding what is needed to re-use samples for international sharing.

Even in the traditional model, concerns remain as to whether participants are sufficiently informed and comprehend the known implications of engaging with research projects. For example, information may be given but not read, or read but not understood; care may or may not be taken with checking comprehension of what is being authorised. Manson and O’Neill [10] describe this as ritualization of consent. If reduced to ritual, consent is meaningful not because it protects autonomy or is informed and voluntary, but it serves merely as a “talisman” that serves principally to authorise research, absolving further duties or liability for researchers once it has been gained.

The process of “consenting”, as it is inappropriately labelled, is now so ubiquitous that for many people it is simply a tick-box exercise. Under such a conception an assumption is made that this is something the clinician or researcher does *to* the participant or patient. It is commonplace to bypass reading the full terms and conditions for a website before ticking the ‘I have read and accept these terms’ box, particularly as this may involve several pages of legal or technical information. This can lead to ritualization as described, and therefore to loss of quality and ethical rigour. In addition to the challenges to informed consent we have briefly noted, the populations from whom consent would be sought in the Athlome Consortium are not patients, who can be considered as highly motivated to participate in research, but healthy athletes. Those consenting to sequencing and/or secondary use of their previously collected samples may potentially uncover disease risk in themselves, or heritable traits that impact their families. This is not a risk that precludes non-clinical genomics, but requires pre-test counselling and considered reporting of results with further support where necessary. This burden on the participant without significant benefit to themselves or to community may weaken the case for a move from informed to broad or other less stringent consent forms in the case of Athlome.

These considerations lead us further to ask whether the traditional model of informed consent is even applicable to biobanks [25, 26], a problem that has been widely discussed in the literature. The possible alternative models of consent to biobank research are discussed here.

## Blanket consent

Blanket consent arises when consent is sought and given for all types of research, or data uses, and no further permissions are sought. It is in effect a “carte blanche”. There is no specificity regarding use and no direct control by the participant over what types of research are carried out, including secondary uses of data. Of the consent models presented, blanket consent is the weakest in terms of protection of individual interests. It may have the property of voluntariness, but not of informedness. A participant retains the right to withdraw, but this is only useful if people know what research is being carried out to withdraw from. Practically, withdrawal is limited in biobanking, as once data is anonymised and linked it is extremely difficult to withdraw. The removal of a person or persons’ information may have also a negative impact on the other samples, as the power of large biobanks is in linking datasets on a large scale. The question of why we might prioritise a person’s autonomy (withdrawing) over another (remaining) where such a withdrawal affects those remaining, deserves further consideration, but will not be discussed in detail here.

Blanket consent may be perceived as justifiable where the risk is minimal, so long as sufficient protections are in place in terms of anonymity and Research Ethics Committee or Internal Review Board oversight, but all forms of consent and governance should reflect societal values. Participants ought to be able to express preferences over the types of research they wish to be a part of. For example, Care. Data is a recent high profile example of a population scale data project whose failure was in no small part due to lack of social approval, or licence [27]. Uncertainty regarding the role and aims of the project, lack of trust inter alia led to failure to gain public support. Research depends on voluntary contribution, based on trust in non-exploitation and public benefit. Where trust is lacking and a project contravenes societal norms or expectations, such as confidentiality of GP medical records, legislating does not replace, nor can it create, social validation [27]. As the name suggests, blanket consent is non specific and does not allow for expression of specific preference. In contrast, broad consent provides some greater control, specifying parameters for research being consented to, but is not as specific as informed consent [26].

## Broad consent

Broad consent is the model currently in use in many biobanks including UKBiobank and is supported in legislative terms by the Human Tissue Act in the UK [26]. It has been argued that this is not a valid form of consent because of its specificity failings, and the inability to meet the basic informational requirements of informed consent. Broad consent can be described as consent to governance, rather than directly to research [14, 26]. This represents a shift away from consent as the individual exercising control, or ‘agent sovereignty’ [28] dependent on the specific project, to placing one’s trust in a governing group and therefore giving control over to that group. In order to qualify as any kind of consent, it is argued that broad consent requires deliberation rather than just information, and as such can still respect personal autonomy via the considered choice to enter a biobank scheme and be governed [14]. In broad consent a person agrees to an outline of research aims, with governance of research activities and dissemination of information to the public overseen by an appropriate governance committee, which should include participant members [26, 29].

## Meta consent

Ploug and Holm [30] propose a consent model that covers research uses throughout the course of a life. They call this “meta consent”. Specific types of research are consented to and can be amended over the life course at the discretion of the participant. The basis of meta consent is that ‘people should be given the opportunity to make choices based on their preferences for how and when to provide consent’ [30, 5]. Such a view places the possibility for processual understandings of consent, but does not force them on the individual participant. Meta consent may protect participant autonomy by enabling a choice over the possible uses of their data. This may be more or less specific. Thus, for example, a participant may say “yes” to cancer research, but “no” to diabetes research. Equally, they may authorise use concerning one particular project (e.g. UK Biobank breast cancer campaign tissue bank) but not others. They may also be invited to elect for their data to have unspecified future use. When a participant is selective about the uses, they should be contacted when new directions or applications for the data are intended. Where a person states a preference for no further contact, they potentially miss the opportunity to take part in research in which they might otherwise have chosen. Where a person chooses to give consent to specific projects only, but requests the opportunity to consider other uses, they will need to be re-contacted for further consent [30]. One model for this is “dynamic consent”.

## Dynamic consent

Dynamic consent involves going back to the participant every time new use of data is proposed [20]. This reflects the ‘gold standard’ of informed consent per research project. It is not, however, without problems. Concerns include ‘consent fatigue’ due to repeated re-contacting, as well as placing an undue burden on participants for little or no personal benefit; for example, Biobank research potentially benefiting future generations, but unlikely to have a direct (therapeutic) impact those participating. This may affect both the numbers of people who sign up initially, and lead to higher attrition over the life of the research biobank. If consent becomes routine, it loses the quality that gives it validity in the first place [30]. Other questions include how distinct the new use has to be from the original use consented to in order to need re-consent, and who decides the boundaries for this. [31] Dynamic consent could be utilised to facilitate a meta consent or broad consent approach. The consent sought on re-contacting might be broad or specific, depending on the context.

## Waived consent

The Council for International Organisations of Medical Sciences (CIOMS) guidelines allow for waiver of informed consent requirement where risk is minimal. There are parallels here with policies of deemed consent for organ donation such as implemented in Wales from 2015, and sometimes referred to as presumed consent. This is of course an oxymoron, as consent is an active verb. One must give one’s consent. If it presumed, it is no longer consent. A person who dies in Wales, having lived therefore at least a year, will have their organs used for transplant unless they have specifically opted out, or a next of kin objects at the point of removal [32]. A point of contrast, however, is the specifiability of the benefit from biobank research exists as a future potential; this may be hard to grasp for participants. By contrast, the result of an increase in organ donation is concrete and auditable. The waiver of consent in organ donation in Wales’ organ procurement policy is ‘soft’ insofar as those aware of the preferences of the deceased may have a right to refuse the procurement, even where the donor did not make a specific objection in their lifetime [32].

It may be inferred that this leeway is indicative of the political unease with harvesting organs without specific authorisation. How far this analogy ought to operate in relation to genetic biobank consent is unclear. And the nature of the object (e.g. an organ versus a much smaller tissue sample or DNA swab) means that the bar should perhaps be lower for biobanking. Waived consent is less problematic in genomic research than organ procurement in this regard. Though this may be harder to justify for sports genomics, particularly studies that are investigating genetic elements of elite performance, where there is a potential commercial gain, than say rare disease research, which has public health benefits. It could be argued that due to the intricate links between clinical research and for example pharmaceutical companies, all research is open to potential commercial profit. It is reasonable to suggest that any ethical framework should in part reflect the current norms of the society in which it operates. This can be challenging enough in one country, but becomes considerably more complex in international governance.

The European Society of Human Genetics suggests diversified informed consent. Consent is required where data are ‘identifiable’ and not where fully anonymised. There are concerns as to how complete any anonymisation or protection can be with improved technologies and increasing linkage of datasets. The complexity of anonymisation and linking technologies also has implication for the practical application of the right to withdraw. UKBiobank sets out tiers of withdrawal starting from “no future use”, to “retain data”, and to “remove and destroy all data with no further contact” [29]. UKBiobank and others make explicit the fact that it will not be possible to remove data from existing or previous studies due to the measures taken to ensure privacy [29]. The right to withdraw is therefore curtailed by the limits of technology and the ways in which the data are captured and used. The governance of data sets is not unified or harmonised in relation to the technologies of consented data.

The right for participants to volunteer yet also withdraw their participation in research has been considered fundamental to ethical research conduct since the Nuremburg trials. A recent challenge to this is the idea of moral obligation to take part in research; the future benefits to society being so great that not only is participating a contribution to the public good, but that those eligible are obliged to do so [33]. As with the move away from informed consent, this seems harder to justify for sports than for other types of research where a direct benefit to clinical populations, as well as society more generally, can be seen. In addition to the perceived differences between sports and clinical research in terms of obvious benefit, the moral duty to take part does not account for future orientated genomics, but is concerned with more general medical research. It seems difficult to justify a moral obligation to participate in such open-ended future uses of biobank research information.

Having discussed the ethical challenges to genomic research biobanks, we now turn specifically to issues arising in the construction of the Athlome Consortium that will need to be addressed before it achieves its stated goals to ‘collectively study the genotype and phenotype data currently available on elite athletes, in adaptation to exercise training (in both human and animal models) and on exercise-related musculoskeletal injuries from individual studies and from consortia worldwide’ [2].

## International regulatory frameworks

The Athlome Consortium, which operates across 6 regions (Africa, Europe, Middle East, North America, Asia and Australasia) faces regulatory and consent issues due to its global nature and to the combining of existing cohorts or ‘legacy collections’ of genetic data. Additionally, the dynamics involved in consent for research, particularly at professional/elite level in sports, may raise specific concerns that although not specific to genomic research, certainly apply. There is not room here to explore these fully, but include concerns regarding potential inducement, coercion, privacy and confidentiality [34].

The individual projects within the Athlome consortium are, therefore, subject to a range of state, national and regional legislation and regulatory frameworks. Such frameworks originate in different jurisdictions and therefore reflect different cultural norms. What has been required as minimum consent in one country may not be the same as another. Regions include the EU, US and UK, which have overarching data sharing laws such as the Data Protection Act, European Data Protection Directive (due into force 2018) and US Common Rule, but often do not have specific biobanking laws [35]. No universal data sharing treaty currently exists, but national laws and international guidelines have their basis in the Universal Declaration of Human Rights [36]. Some countries differentiate between international sharing of samples and data; for example, in Taiwan sample sharing is not allowed at all, but data may be. Others may not differentiate explicitly, but have limitations on sharing; for example, in Demark sharing is allowed with participant consent and a Danish collaborator [35].

In the Athlome Consortium, projects work across the regions mentioned, but also in areas where ethical and regulatory frameworks are less developed. It is a huge challenge to respect cultural norms and avoid imposing one dominant region’s framework onto all partners, whilst protecting the interests of both the individual participant and the common interests of the research groups. The latest Council of Europe Recommendation (CM/Rec(2016) 6) places emphasis on interoperability and international cooperation in biobank research and gives some guidance on governance, oversight and transborder flows; for example, leaving open the methods by which this is to be achieved [37].

Although much research in biobanks is prospective, existing samples may be used for new research purposes and may require retrospective consent. In retrospective studies, there is a likelihood of samples having been consented to differing standards, or potentially not consented at all. Some retrospective biobanks may rely on ‘old consent’ from many years previous [16], which may not reflect the current thinking of the individual donor, and is unlikely to have included the uses now being proposed. This poses the question of how to ensure quality of consent whilst not impeding research due to the time and cost of procedures. The consent models discussed above may offer some recourse to the problem, but do not solve it. Where secondary research aims are not consistent with those for whch consent was originally given, consent for the new uses should be gained, with the exception of fully anonymised materials. If blanket consent is used then this aspect is vitiated, as the initial consent process covers all possible future uses. It is not possible to re-contact the donors of permanently de-identified materials, and regulation does not require further consent for secondary use of such materials. The secondary use of existing materials is a key challenge for Athlome and other similar collaborative projects; not only because of new uses, but also because of the differing standards and types of consent given by each partner’s participants in the first place.

Collaboration brings rewards in terms of increased dataset size, diversity and analytical power, plus reduction in costs and need for replication. Yet, with those rewards come challenges for consent and governance. Biobanks are governed by the legislative jurisdiction in which they are physically located. They may also be governed by regional legislation and governance frameworks, for example, EU Law, CIOMS, Council of Europe Recommendations. Within a specific country there may also be state and federal legislation regarding data sharing and biobank research, for example, in the US, Germany. In the UK the Data Protection Act 1998 covers the whole union, but human tissue legislation operates differently with regard to consent for research using human tissue samples between Scotland [38] and the rest of the UK, leading to inconsistency in the law applicable to biobank research within the UK.

In 2013 the European Research infrastructure Consortium (ERIC) for biobanking was established. This group develops standards for biobanks, but does not make significant contributions to addressing the ‘diverse regulatory framework for biobanking in the EU and the Member States’ [39]. Nevertheless, by definition, ERIC is limited in scope to Europe. Coalitions such as GA4GH and P^3^G have also produced instruments such as the Framework for Responsible Sharing of Genomic and Health-Related Data with the aim of providing means to ethically share data in a manner practical for all concerned [24]. These offer a practical starting point for Athlome to develop governance to be tailored to their specific range of needs.

Whilst the EU makes law, and Member States draw down such laws into their legislature, it is the responsibility of each Member State to implement that law. Member States retain sovereignty both in the application of laws and in how they (States) relate to each other [39]. Frameworks such as the ERICs are closely tied to EU Law, but the national law of the country in which a biobank is located will be the first legal control. This regulatory heterogeneity creates uncertainty, and is a barrier to effective collaboration [1].

The complexity of sharing data across multiple unrelated jurisdictions, such as in the Athlome Consortium, is considerable. There is, therefore, a need to develop supporting ethical principles alongside a governance framework that encompasses and respects the differing norms of the countries involved. It is not appropriate to simply impose an existing legislation or framework onto the rest. Mallette et al. [1] suggest a model governance framework that addresses some of the problems raised above, as part of P^3^G. The setting out of ethical principles, as opposed to a top-down prescriptive approach, aims to support common regulation by providing a shared base from which to develop more sophisticated governance. Proactively working to create foundational principles that guide law and governance, can provide greater clarity and reduce the amount of reactive legislation necessary to catch up to technology.

In Europe, the regulation for genetic and genomic research is more advanced, but Athlome is global. Athlome might be guided by the work of P^3^G, GA4GH, the TRUST Project, for example, in developing a central ethical framework that allows for international data sharing and sets out protocols for secondary use of samples and data. In doing so we must be careful to take a developmental approach, allowing flexibility to react quickly as needed and not ‘create problems’ in an attempt to be proactive. Operating with local codes and ‘soft law’ rather than through universal legislature may be a way to facilitate such an approach [35]. Seeking commonalities is the basis for developing harmonized governance that can better accommodate and respect difference whilst providing a way forward for collaborative biobank research. One method proposed by GA4GH to support work across very different normative settings is to assess existing governance; for example, REC provision ‘essential elements’ in order that agencies can have confidence in each other’s systems [24].

Data security is of concern to biobank projects, both at individual and dataset level. Maintaining data security can be linked to preserving privacy and confidentiality by using sophisticated coding and anonymisation techniques. The use of ‘trusted third parties’ (TTPs) is common in big data banks, such as the SAIL (Secure Anonymised Information Linkage) databank, UK [40]. A central TTP that holds the identifiers/codes for all Athlome data could form part of a governance framework based on ethical principles that covers all of the partners in the consortium. One aspect of data security worth considering, but beyond the scope of this paper, is the impact of different surveillance legislations e.g. Investigatory Powers Act 2016 [41] concerning who is able to access data and communication thereof, and for what purposes.

## Conclusion

While consent is one of many ethical concerns around biobank research, it is undeniably a central one. We have briefly but critically reviewed the main consent models either in use, or potentially appropriate for biobanking. For researchers, the more open the consent, the more potential research uses are available to be explored. Conversely, the more restricted the consent, the fewer possible uses without seeking further consent. The form of consent to be used should fit the nature and purposes of the research. Anonymity and confidentiality notwithstanding, a high degree of latitude might be thought to compromise individual consenters’ interests.

With the exception of blanket consent, it seems clear that each of the consent types is viable on its own terms as part of an ethical model for biobank project development. The choice of consent model ought to some extent depend on, and reflect, the nature of the research being undertaken. Some flexibility seems unavoidable given that biobank development is in relative infancy compared other forms of research programmes. It appears unjustifiable to prescribe too closely the forms of consent in such a rapidly developing and complex field.

Athlome and other collaborative genomics projects face challenges around joining data from previous projects. This should be done only where it is broadly consistent with the aims to which participants originally consented. Regulation is rendered extremely complex by the multiplicity of sources of data, and it has been noted that a ‘one size fits all’ all policy is a chimera in the case of international biobank or genomic research. Ethical principles that guide tailored frameworks, rather than a single set of regulations, may be more helpful in conjunction with the use of TTPs as a practical safeguard for both privacy and security.

We have not discussed commercial uses of sports genomics data or the closure of biobanks, but these are areas that merit further consideration, and should be part of any future framework. [42] Fuller consideration of the other ethical concerns relating to biobank research should include reporting of results to participants (specifically Incidental or Secondary Findings), deletion of data and the ‘right to withdraw’, and ownership of results and potential gains from results of research. Transparency and accountability as well as inclusive public engagement is vital from before the start of participant recruitment and continuing throughout the lifespan of a biobank or related research project [1].

These considerations should contribute to the nascent understanding of ethical and regulatory practices by considering how to meet the challenges of governance in the context of the Athlome Project and projects like it in the ethics of sports genomics, which is still in its infancy in comparison to medical or clinical ethics. For these reasons as well as those that apply to genomic research more generally, it is important that new biobank research groups such as Athlome embed robust ethical and regulatory practice in their structures from the outset. The careful consideration of underlying norms that inform how consent and other challenges for governance are conceptualized, and how data can be shared in a manner that is both ethical and supportive of the research, is key to developing and sustaining their success.
